# The radiosensitising effect of gemcitabine and its main metabolite dFdU under low oxygen conditions is *in vitro* not dependent on functional HIF-1 protein

**DOI:** 10.1186/1471-2407-14-594

**Published:** 2014-08-16

**Authors:** An Wouters, Bea Pauwels, Natalie Burrows, Marc Baay, Vanessa Deschoolmeester, Trung Nghia Vu, Kris Laukens, Paul Meijnders, Dirk Van Gestel, Kaye J Williams, Danielle Van den Weyngaert, Jan B Vermorken, Patrick Pauwels, Marc Peeters, Filip Lardon

**Affiliations:** Center for Oncological Research Antwerp, University of Antwerp, Universiteitsplein 1, 2610 Wilrijk, Belgium; Hypoxia and Therapeutics Group, University of Manchester, Oxford Road, Manchester, M13 9PT UK; Cambridge Institute for Medical Research, University of Cambridge, Addenbrooke’s Hospital, 4.17 Wellcome Trust/MRC Building, Hills Road, Cambridge, CB2 0XY UK; Biomedical Informatics Research Center Antwerp (Biomina), University of Antwerp, Middelheimlaan 1, 2020 Antwerpen, Belgium; Department of Radiotherapy, University Radiotherapy Antwerp (URA), Lindendreef 1, 2020 Antwerp, Belgium; Department of Oncology, Antwerp University Hospital, Wilrijkstraat 10, 2650 Edegem, Belgium; Department of Pathology, Antwerp University Hospital, Wilrijkstraat 10, 2650 Edegem, Belgium

**Keywords:** Hypoxia, Radiosensitisation, Gemcitabine, dFdU, HIF-1

## Abstract

**Background:**

Regions within solid tumours often experience oxygen deprivation, which is associated with resistance to chemotherapy and irradiation. The aim of this study was to evaluate the radiosensitising effect of gemcitabine and its main metabolite dFdU under normoxia versus hypoxia and to determine whether hypoxia-inducible factor 1 (HIF-1) is involved in the radiosensitising mechanism.

**Methods:**

Stable expression of dominant negative HIF-1α (dnHIF) in MDA-MB-231 breast cancer cells, that ablated endogenous HIF-1 transcriptional activity, was validated by western blot and functionality was assessed by HIF-1α activity assay. Cells were exposed to varying oxygen environments and treated with gemcitabine or dFdU for 24 h, followed by irradiation. Clonogenicity was then assessed. Using radiosensitising conditions, cells were collected for cell cycle analysis.

**Results:**

HIF-1 activity was significantly inhibited in cells stably expressing dnHIF. A clear radiosensitising effect under normoxia and hypoxia was observed for both gemcitabine and dFdU. No significant difference in radiobiological parameters between HIF-1 proficient and HIF-1 deficient MDA-MB-231 cells was demonstrated.

**Conclusions:**

For the first time, radiosensitisation by dFdU, the main metabolite of gemcitabine, was demonstrated under low oxygen conditions. No major role for functional HIF-1 protein in radiosensitisation by gemcitabine or dFdU could be shown.

## Background

Regions within solid tumours often experience mild to severe oxygen deprivation (hypoxia) owing to aberrant vascular structure and function. Multiple clinical studies have documented the importance of hypoxia and anoxia (an absence of oxygen) in determining local tumour control in radiotherapy. In addition to the direct role of oxygen in generating radiation-induced DNA damage, the biological effects of hypoxia on tumour cells can also modulate their response to therapy [1, 2]. One major transcription factor involved in the cellular response to reduced oxygen conditions is hypoxia inducible factor 1 (HIF-1). This heterodimeric transcription factor is formed by the association of an oxygen-regulated HIF-1α subunit with a constitutively expressed HIF-1β subunit. As HIF-1 modulates many cellular processes, including proliferation, apoptosis, metabolism and the tumour vasculature, it has been reported that HIF-1 has divergent effects on tumour radiosensitivity, which might cause tumours to become more or less radiosensitive [3].

Among the most potent (normoxic) radiosensitisers currently available are antimetabolites. Gemcitabine (2’ ,2’-difluorodeoxycytidine, dFdC) is a synthetic pyrimidine nucleoside analogue clinically active against a broad spectrum of solid tumours. Intracellularly, the diphosphate (dFdCDP) and triphosphate (dFdCTP) forms of the drug are responsible for the cytotoxic effects, via inhibition of ribonucleotide reductase and by incorporation into the DNA, leading to chain termination, respectively. In addition to its cytotoxic effect, gemcitabine has potent radiosensitising properties, shown in both preclinical and clinical studies [4]. Current evidence suggests that accumulation in the S phase of the cell cycle, depletion of dATP pools, reduction of apoptotic threshold, inhibition of DNA synthesis and reduction of DNA repair may contribute to, or might even be essential for gemcitabine-mediated radiosensitisation [5].

Following intravenous administration of gemcitabine, the drug rapidly undergoes deamination to its main metabolite, 2’ ,2’-difluorodeoxyuridine (dFdU), resulting in a plasma half-life of gemcitabine of only eight minutes [6]. In contrast, the half-life of dFdU is greater than 14 h, yielding elevated dFdU plasma concentrations for a prolonged period of time (>24 h) at levels known to cause growth inhibition. Importantly, although dFdU has limited cytotoxic activity, it has been demonstrated that it causes a clear concentration- and schedule-dependent radiosensitising effect *in vitro* and potentially contributes to the potent radiosensitising properties of gemcitabine in the clinic [7].

Thus far, few preclinical studies have focused on the outcome of chemoradiation treatments under hypoxia, and on the potential impact of functional HIF-1 on the radiosensitising effect of cytotoxic agents. The molecular basis of hypoxia-mediated chemotherapy and radiotherapy failure indeed has only recently been reported. In these studies, a contribution of HIF-1 to drug resistance has been observed in a wide spectrum of neoplastic cells and many signalling pathways, including PI3K, MAPK, HER2, EGFR and COX2, are reported to induce chemoresistance through HIF-1 activity [8–11].

Concerning gemcitabine, it has recently been observed that this drug radiosensitises both p53 wild type and p53 deficient non-small cell lung cancer cells under hypoxia [12]. Although it was described that gemcitabine did not affect tumour oxygenation or HIF-1α levels in HCT116 xenografts [13], it has also been reported that gemcitabine inhibited HIF-1α induction in A549 cells exposed to the hypoxia mimetic agent DFX [14]. In contrast, a more recent study showed gemcitabine-induced activation of HIF-1α in normoxic pancreatic cancer cells [15]. In order to further elucidate whether or not the HIF-1 transcription factor is involved in the retained radiosensitisation by gemcitabine under low oxygen conditions, in the present study, we evaluated the impact of hypoxia on radiosensitisation by gemcitabine and dFdU in three isogenic breast adenocarcinoma cell lines differing in HIF-1 status.

## Methods

### Cell culture

The human tumour cell lines included were MDA-MB-231 (breast adenocarcinoma; wild type (wt) HIF-1) and the sublines MDA-MB-231 dnHIF (dominant-negative HIF-1α; HIF-1 activity inhibited) and MDA-MB-231 empty vector control (EV; functional HIF-1). MDA-MB-231 sublines were constructed as described previously [16], resulting in MDA-MB-231 cells stably expressing dnHIF tagged with enhanced green fluorescence protein (eGPF) or eGFP alone (MDA-MB-231 dnHIF and MDA-MB-231 EV, respectively). The dnHIF construct inhibits HIF-1 activity by competing with endogenous HIF-1α for interaction with HIF-1β and DNA binding; it is however likely that non-canonical regulation by HIF-1 is not inhibited, since the dnHIF construct is identical to endogenous HIF-1α except for loss of the oxygen-dependent degradation domains and DNA-binding domains. All cell lines were free from mycoplasma contamination. Cultures were maintained in exponential growth in a humidified 5% CO_2_/95% air atmosphere at 37°C (normoxia).

### Oxygen conditions

Hypoxia (<0.1% O_2_) was achieved in a Bactron IV anaerobic chamber (Shel Lab, Cornelius, USA), as described previously [17]. Hypoxic incubation was initiated after cells had been cultured under normoxia overnight, allowing attachment to culture dishes.

### Western blot analysis

Cells were placed under normoxia or hypoxia for 18 h, yielding a robust induction of the expression of HIF-1α and HIF-1-induced downstream targets. Subsequently, cells were lysed and protocols were used as previously described [18]. In short, cells were lysed in 100 μl lysis buffer (10 mM Tris (pH 7.4), 150 mM NaCl, 1 mM EDTA, 1 mM EGTA, 50 mM NaF, 1 mM sodium orthovanadate, 1% Triton X-100 v/v, 0.5% Nonidet P-40 v/v, 2 mM leupeptin, 0.15 mM aprotinin, 1.46 mM pepstatin, 1 mM phenylmethansulfonyl fluoride). For western blot analysis, proteins (20 μg/lane) were resolved on a 7.5% SDS-PAGE gel and electrotransferred onto a polyvinylidene fluoride (PVDF) membrane (Millipore, Schwalbach, Germany) using standard procedures. After blocking with 5% non-fat dry milk w/v in PBS-T (137 mM NaCl, 2.7 mM KCl, 4.3 mM di-sodiumhydrogenphosphate, 1.4 mM potassium-di-hydrogenphosphate, 0.1% Tween20 v/v) overnight at 4°C, the blot was probed with primary antibodies (mouse monoclonal anti-HIF-1α (BD Transduction Laboratories, Oxford, UK); monoclonal anti-CA9 (clone M75; kindly provided by Dr. Jaromir Pastorek, Bratislava, Slovakia)). The blot was then reacted with suitable secondary alkaline phosphatase-conjugated antibodies (Dianova, Hamburg, Germany), followed by detection of the protein with CDP-Star chemiluminescent reagent (Applied Biosystems, Foster City, USA). Finally, the blot was stripped and reprobed for β-actin (mouse monoclonal anti-β-actin, Sigma-Aldrich, Dorset, UK) to ensure equal loading and transfer of proteins.

### Immunofluorescence

To assess dnHIF-mediated inhibition of CA9, cells were cultured on sterile coverslips under normoxia or hypoxia for 18 h, fixed for 10 min and permeabilised. Cells were incubated with a mouse monoclonal anti-human CA9 antibody (clone M75) for 1 h at 37°C, washed and incubated with a secondary Alexaflour antibody (Life Technologies, Paisley, UK) for 45 min at 37°C. Cells were then washed and nuclei were counterstained with 4’ ,6-diamidino-2-phenylindole (DAPI) and mounted in DAKO fluorescent mounting media (DAKO, Cambridgeshire, UK). Relative localisation of green (eGFP; to identify eGFP in empty vector cells and the dnHIF construct in dnHIF cells), red (for CA9) and blue (for nuclei) fluorescence was analysed with a snapshot wide-field fluorescence microscope and MetaView software.

### HIF-1 activity assay

HIF-1α activity was assessed as described previously [18]. Briefly, cells were transfected with an adenovirus containing trimers of the LDH-A HRE linked to luciferase and were exposed to hypoxia for 18 h. Afterwards, cells were lysed, luciferase activity per μg protein was calculated and activity of MDA-MB-231 dnHIF cells was normalised to MDA-MB-231 EV control cells.

### Vascular endothelial growth factor ELISA

Cells were incubated under normoxia or hypoxia for 18 h, then the media was removed for analysis of secreted VEGF levels as previously described [18]. The concentration of secreted VEGF was determined using a Duoset Human VEGF ELISA kit (R&D Systems, Abingdon, UK) according to the manufacturer’s instructions, and corrected to the amount of protein within the cell cultures from which the medium was taken.

### Human hypoxia signalling pathway PCR array

After 24 h incubation under hypoxia, total RNA samples were isolated from 4.10^6^ normoxic and hypoxic cells, using RNeasy® Mini Kit (Qiagen, Venlo, The Netherlands). The concentration of extracted RNA (A_260_/A_280_ ratio) and purity (A_260_/A_230_ ratio) were measured by Nanodrop ND-1000 spectrophotometer (Isogen, Sint-Pieters-Leeuw, Belgium). The quality of isolated RNA was confirmed by capillary electrophoresis with an Agilent 2100 Bioanalyzer (Agilent Technologies, Amstelveen, The Netherlands). One μg RNA was reversed transcribed using ReactionReady First Strand cDNA Synthesis Kit (Qiagen) in accordance with the manufacturer’s instructions. The relative expression of 84 genes related to the hypoxia signalling pathway was assessed by use of the Human Hypoxia Signalling Pathway PCR Array (Qiagen) and the RT^2^ Real-time SYBR Green/Rox PCR Master mix kit (Qiagen).

### Chemoradiation clonogenic assay

MDA-MB-231 cells were exposed to normoxia or hypoxia and treated with 0–8 nM gemcitabine or 0–4 μM dFdU for 24 h immediately before and during irradiation (0–8 Gy, room temperature, XRAD320 irradiator (Precision X-Ray, North Branford, USA)). Treatment schedule and concentrations of gemcitabine and dFdU were chosen based on previous results [12, 19]. To irradiate cells under hypoxia, custom-made airtight Perspex shells, preincubated in the anaerobic chamber overnight, were used [17]. Immediately following irradiation, hypoxic cells were reoxygenated and all cells were washed with drug-free medium. Unirradiated control cells were handled identically to treated cells. Following an 8-day incubation period, cells were stained with crystal violet and colonies (>50 cells) were counted as described previously [20].

### Analysis of the cell cycle distribution

Cells were incubated with 0–8 nM gemcitabine or 0–4 μM dFdU for 24 h, under normoxia or hypoxia. Cell cycle distribution was monitored according to the Vindelov method, as previously described [12]. Samples were analysed using a FACScan flow cytometer (Becton Dickinson). Histograms of DNA content were analysed using WinMDI software to determine the fractions in each phase of the cell cycle (G_0_/G_1_, S and G_2_/M).

### Statistical analysis

All experiments were performed independently at least three times, and each experiment comprised at least two parallel samples. Results, if not otherwise stated, are presented as mean ± standard deviation (SD). Statistical differences were evaluated with two-sided two-sample t-tests, one-way ANOVA or two-way ANOVA, using SPSS v16.0 software. Two-way ANOVA was used to study the influence of oxygen tension, HIF functionality and treatment with gemcitabine, dFdU and/or irradiation on the outcome parameter (i.e. cell survival or cell cycle distribution). *Post hoc* comparisons revealed which groups differed significantly from one another. P values less than 0.05 were considered to be statistically significant.

For irradiation experiments, survival rates were calculated as [mean plating efficiency of treated cells/mean plating efficiency of control cells] × 100%. Radiation survival curves were fitted according to the linear-quadratic model using WinNonlin (Pharsight, Mountain View, USA) with survival = exp(-αD-βD^2^). The following parameters were calculated: ID_50_ (radiation dose producing a surviving fraction of 50%); SF_2_ (surviving fraction at 2 Gy); and mean inactivation dose (MID). The oxygen enhancement ratio (OER) was determined by dividing ID_50_ under hypoxia by ID_50_ under normoxia. The dose enhancement factor (DEF) was calculated as ID_50_ for control, untreated cells divided by ID_50_ for treated cells.

Possible synergism between gemcitabine or dFdU and radiation was determined by calculation of the combination index (CI) using CalcuSyn software (Biosoft, Cambridge, UK). CI < 1.0, CI = 1.0 and CI > 1.0 indicated synergism, additivity or antagonism, respectively.

To analyse the PCR array data, relative changes in gene expression were calculated using the ΔΔC_t_ method. Based on the geNorm algorithm, four endogenous control genes were selected for normalisation. Each replicate cycle threshold (C_t_) was normalised to the average C_t_ of the four endogenous controls on a per plate basis and mRNA expression levels were presented as fold changes.

## Results

### Validation of stable transfection of MDA-MB-231 cells with dnHIF

**Figure 1:**
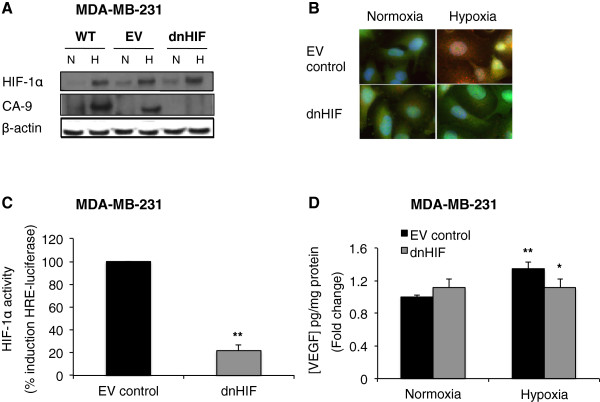
**dnHIF expression reduces expression of HIF-1 target proteins (CA-9 and VEGF) and HIF-1α activity. A**. Western blot analysis of HIF-1α, dnHIF and CA9 protein level in MDA-MB-231 wt, EV and dnHIF cells. Cells were exposed to normoxia (N) or hypoxia (H) for 18 h. β-actin detection served as the loading control. **B**. Fluorescent images of MDA-MB-231 EV and dnHIF cells stained with eGFP/dnHIF (green), CA9 (red) and DAPI (blue). Cells were exposed to normoxia or hypoxia for 18 h. **C**. HIF-reporter assay in MDA-MB-231 cells stably-expressing dnHIF or empty vector (EV) control following 18 h hypoxic exposure. Luciferase activity per μg protein was calculated and activity was normalised to EV control cells (**: p < 0.01 in dnHIF vs. EV cells). All results are from at least 3 independent experiments ± SD. **D**. VEGF levels in media taken from dnHIF and EV control cells in normoxia/hypoxia for 18 h. VEGF media levels were normalised to mg protein within the cell cultures from which the media were taken. Hypoxia significantly increased VEGF expression in control MDA-MB-231 EV cells (**: p < 0.01 in hypoxic vs. normoxic cells) but not in dnHIF cells. Most importantly, inhibition of HIF by use of a dominant-negative protein variant (dnHIF) inhibited hypoxia-induced VEGF expression (*: p < 0.05 in dnHIF vs. EV cells). All results are from at least 3 independent experiments ± SD.

Exposure to 18 h hypoxia induced expression of HIF-1α and its downstream target carbonic anhydrase 9 (CA9) in MDA-MB-231 wt and EV cells (Figure 1A). In MDA-MB-231 dnHIF cells, HIF-1α expression was detected in hypoxic cells exposed to reduced oxygen levels too. However, due to the presence of the dnHIF protein, CA9 expression remained markedly lower in comparison with the HIF-1α proficient cell lines. As shown in Figure 1B, the dnHIF construct was localised to the nucleus and was expressed independently of oxygen availability. Transfection with the control vector (EV) resulted in eGFP expression that was confined to the cytoplasm. Moreover, the immunofluorescence images clearly showed induction of CA9 in the EV cells under hypoxic conditions, while CA9 staining was absent in hypoxic dnHIF cells.

In order to quantitatively evaluate the impact of dnHIF on HIF-1 function, an adenoviral-based HIF-1α reporter gene assay showed that HIF-activity was significantly inhibited (p < 0.01) in cells expressing the dnHIF protein (Figure 1C). Furthermore, hypoxia-induced VEGF secretion was significantly lower (p < 0.05) in dnHIF versus EV cells (Figure 1D).

### Human hypoxia signalling pathway PCR array

**Figure 2:**
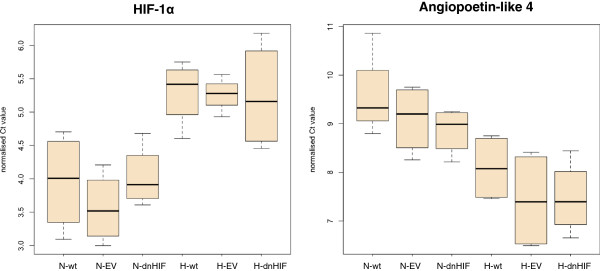
**mRNA expression of hypoxia-related genes under normoxia versus hypoxia in MDA-MB-231 cells.** Normalised C_t_ values for HIF-1α (left) and angiopoetin-like 4 (right), as analysed by the Human Hypoxia Signalling Pathway PCR array. Boxplots present the normalised C_t_ values for MDA-MB-231 wt, EV and dnHIF cells exposed to normoxic (N) or hypoxic (H) conditions for 24 h. A higher C_t_ value corresponds to lower mRNA expression in the biological sample.

Out of 84 genes related to the hypoxia signalling pathway, 11 genes showed a more than two-fold up- or downregulation in mRNA levels between normoxic and hypoxic conditions (Table 1). For HIF-1α, a decreased mRNA expression level was observed after 24 h hypoxia (Figure 2). Statistical analysis revealed no differential expression profile between MDA-MB-231 wt and EV versus MDA-MB-231 dnHIF cells for the hypoxia-related genes included in the PCR array. A significant difference in expression level under normoxia versus hypoxia was noticed for HIF-1α and angiopoetin-like 4 in MDA-MB-231 wt and EV cells (p < 0.042); in MDA-MB-231 dnHIF cells, this was only detected for angiopoetin-like 4 (p = 0.026). Significance was however lost when p-values were adjusted for multiple comparisons according to the Benjamini-Hochberg procedure.

**Table 1:** **mRNA expression profiles of hypoxia-related genes in MDA-MB-231 wt, EV and dnHIF cells**

Symbol	Description	Fold up- or downregulation under hypoxia versus normoxia		
		MDA-MB-231 wt	MDA-MB-231 EV	MDA-MB-231 dnHIF
ADM	Adrenomedullin	2.18	1.73	-1.00
ANGPTL4	Angiopoetin-like 4	2.72	3.19	2.65
BHLHE40	Basic helix-loop-helix family, member e40	1.56	2.18	2.16
CASP1	Caspase-1	-1.44	-2.03	-1.67
HIF-1α	Hypoxia inducible factor 1, alpha subunit	-2.61	-3.26	-2.29
HIF-3α	Hypoxia inducible factor 3, alpha subunit	-2.25	-1.71	-1.68
IPCEF1	Interaction protein for cytohesin exchange factors 1	-3.75	-1.51	4.48
LCT	Lactase	-1.02	-2.39	-1.73
LEP	Leptin	-1.96	-1.04	-4.06
MT3	Metallothionein 3	1.52	1.06	2.01
SLC2A4	Solute carrier family 2 (facilitated glucose transporter), member 4	-2.10	-1.16	-1.38

Only genes with a more than two-fold up- or downregulation under hypoxia versus normoxia are presented. (-) represents downregulation. Cells were exposed to normoxia/hypoxia for 24 h (see Methods for full details). All results are from at least 3 independent experiments.

### Cell survival after treatment with gemcitabine or dFdU plus radiation

**Figure 3:**
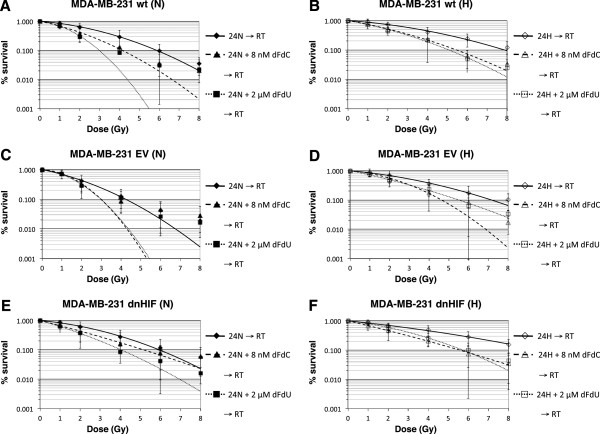
**Clonogenic survival after treatment with gemcitabine or dFdU plus radiation under normoxia versus hypoxia.** Radiation dose response curves of MDA-MB-231 wt **(A, B)**, MDA-MB-231 EV **(C, D)** and MDA-MB-231 dnHIF **(E, F)** cells after 24 h treatment with 8 nM gemcitabine (dFdC) or 2 μM dFdU under normoxia (N) or hypoxia (H), immediately followed by radiation (RT) and reoxygenation. Survival curves were corrected for the cytotoxic effect of gemcitabine or dFdU alone and/or for loss of clonogenic capacity induced by exposure to hypoxia (see Table 2). All results are from at least 3 independent experiments ± SD.

A concentration-dependent radiosensitising effect was observed for gemcitabine and dFdU (Figure 3, Table 2), yielding a moderately synergistic to synergistic interaction between gemcitabine and irradiation. Radiosensitisation was similar under normoxia and hypoxia (p = 0.477 for gemcitabine, p = 0.563 for dFdU). Moreover, the dose enhancement factor was not significantly influenced by the cell line used (p = 0.736 for gemcitabine, p = 0.832 for dFdU) and a similar degree of radiosensitisation was observed with gemcitabine and dFdU in MDA-MB-231 wt, EV and dnHIF cells. Cell survival was significantly influenced by the concentration of gemcitabine or dFdU, dose of radiation, and oxygen tension (p ≤ 0.001). While treatment with gemcitabine or dFdU and exposure to hypoxia both had a significant impact on ID_50_, MID and SF_2_, no significant difference for these radiobiological parameters was seen between EV versus dnHIF cells.

**Table 2:** **Radiobiological parameters for the combination of gemcitabine or dFdU with irradiation under normoxia or hypoxia**

Condition	OER	SD	DEF	SD	CI	SD		ID _50_	SD		MID	SD		SF _2_	SD		% survival	SD	
**MDA-MB-231 wt**																			
24 N → RT								2.83	1.03		3.30	1.06		65.61	21.99		100	0	
24 N + 8 nM dFdC → RT			1.70	0.96	0.865	0.175	*	1.70	0.72	††	2.00	0.76	††	37.48	17.47	††	60	27	††
24 N + 2 μM dFdU → RT			2.18	0.57	0.701	0.214	*	1.46	0.22	††	1.62	0.28	††	29.63	9.83	††	41	12	††
24 H → RT	1.43	0.61						3.67	1.03		4.14	0.98		72.96	16.96		93	11	
24 H + 8 nM dFdC → RT			1.59	0.46	0.683	0.243	¶	2.37	1.32	††	2.51	1.02	††	58.92	17.91		60	25	††
24 H + 2 μM dFdU → RT			2.34	0.71	0.687	0.285	¶	1.87	0.59	††	2.34	0.32	†,††	49.39	15.89		44	8	††
**MDA-MB-231 EV**																			
24 N → RT								2.35	1.29		2.65	1.37		53.68	34.97		100	0	
24 N + 8 nM dFdC → RT			1.70	0.82	0.932	0.273		1.59	0.66		1.61	0.24		28.02	8.46		39	9	††
24 N + 2 μM dFdU → RT			1.74	1.06	0.839	0.231	*	1.80	0.75		2.05	0.72		40.16	26.53		45	23	††
24 H → RT	1.57	0.76						3.41	1.78		4.42	1.81		68.53	32.22		92	36	
24 H + 8 nM dFdC → RT			1.77	0.85	0.381	0.023	¶	2.00	0.47		2.56	1.04		52.56	22.09		40	30	††
24 H + 2 μM dFdU → RT			2.35	1.16	0.711	0.245	*	2.01	0.45		2.59	0.71		48.64	7.71		49	24	
**MDA-MB-231 dnHIF**																			
24 N → RT								3.02	1.42		3.46	1.73		69.79	38.04		100	0	
24 N + 8 nM dFdC → RT			1.50	0.75	0.761	0.182	*	1.82	1.18		2.25	1.16		42.90	26.39		30	15	††
24 N + 2 μM dFdU → RT			2.45	0.67	0.818	0.274	*	1.86	0.99		2.07	1.13		45.32	33.00		61	15	††
24 H → RT	1.43	0.54						3.80	1.40		4.93	1.47		81.34	35.51		97	19	
24 H + 8 nM dFdC → RT			2.04	0.93	0.587	0.280	¶	1.90	0.86	††	1.97	0.69	††	42.62	20.43	††	32	15	††
24 H + 2 μM dFdU → RT			2.08	0.52	0.798	0.311	*	2.54	0.77		2.85	0.83	††	59.16	14.76		63	16	††

dFdC: gemcitabine; RT: irradiation; N: normoxia; H: hypoxia; SD: standard deviation; OER: oxygen enhancement ratio; DEF: dose enhancement factor; CI: combination index, ID_50_: radiation dose producing a surviving fraction of 50%; MID: mean inactivation dose (MID), SF_2_: surviving fraction at 2 Gy; % survival: representing the cytotoxic effect of gemcitabine (dFdC) or dFdU and/or hypoxia alone, 0 Gy.

†: p < 0.05 vs. corresponding normoxic condition (same treatment, same cell line); ††: p < 0.05 vs. corresponding untreated condition (same pO_2_, same cell line).

*: moderate synergism (0.7 > CI > 0.9); ¶: synergism (CI < 0.7).

All results are from at least 3 independent experiments ± SD.

### Cell cycle analysis after 24 h treatment with gemcitabine or dFdU

**Figure 4:**
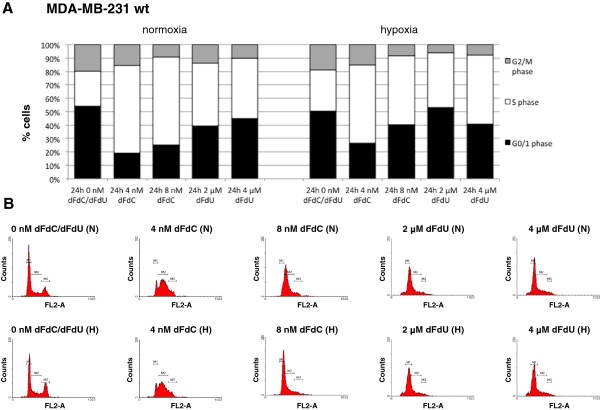
**Cell cycle analysis after 24 h treatment with gemcitabine or dFdU under normoxia versus hypoxia. A**. Cell cycle distribution of MDA-MB-231 wt cells. Cells were treated with 0, 4 or 8 nM gemcitabine (dFdC) or 2 or 4 μM dFdU for 24 h under normoxia or hypoxia. All results are from at least 3 independent experiments. **B**. Cell cycle histogram of MDA-MB-231 wt cells after 24 h incubation with 0, 4 or 8 nM gemcitabine (dFdC) or 2 or 4 μM dFdU for 24 h under normoxia (N) or hypoxia (H). FL2-A = DNA content; M1 = G_0/1_ phase; M2 = S phase; M3 = G_2_/M phase.

In contrast to previous findings in other cell lines [17], exposure of MDA-MB-231 cells to hypoxia did not induce a significant increase in the percentage of G_0/1_ cells (p = 0.213) (Figure 4). The number of cells in S phase was significantly influenced by the concentration of gemcitabine or dFdU (p < 0.001). Gemcitabine and dFdU caused a S phase block in MDA-MB-231 wt, EV and dnHIF cells, both under normoxia and hypoxia (Table 3). As shown on the DNA histograms (Figure 4B), the cell cycle arrest was clearly dependent on the concentration of the drug and shifted from a S phase block to an early S phase block, near the G_1_/S border, with increasing concentrations of gemcitabine and dFdU. *Post hoc* analysis revealed no significant difference in the percentage of G_0/1_, S or G_2_/M phase cells between MDA-MB-231 EV and MDA-MB-231 dnHIF at any condition tested, suggesting that the cell cycle perturbations were not dependent on functionality of HIF-1α.

**Table 3:** **Cell cycle distribution after 24 h gemcitabine or dFdU under normoxia or hypoxia**

Condition	G _0/1_ phase			S phase			G _2_/M phase		
	Mean	SD		Mean	SD		Mean	SD	
**MDA-MB-231 wt**									
0 nM dFdC/dFdU (N)	51.36	7.38		24.85	3.39		18.79	4.90	
8 nM dFdC (N)	25.76	8.82	*	67.73	6.35	*	9.65	1.66	*
2 μM dFdU (N)	37.25	8.74	*	44.28	18.19	*	13.07	8.33	
0 nM dFdC/dFdU (H)	48.61	6.04		29.69	7.94		18.41	8.85	
8 nM dFdC (H)	41.95	17.94		53.28	17.17	*	8.81	1.43	*
2 μM dFdU (H)	49.31	11.21		37.79	12.98		5.46	0.22	
**MDA-MB-231 EV**									
0 nM dFdC/dFdU (N)	44.02	9.86		25.31	5.77		16.31	2.50	
8 nM dFdC (N)	43.97	6.20		29.82	7.59		13.53	4.80	
2 μM dFdU (N)	39.05	15.49	*	42.19	19.60	*	8.72	2.45	
0 nM dFdC/dFdU (H)	51.31	11.10		27.28	9.94		10.17	4.03	¶
8 nM dFdC (H)	52.53	13.96		28.11	13.79		8.57	2.37	¶
2 μM dFdU (H)	50.51	14.73		33.15	19.84		6.56	2.31	
**MDA-MB-231 dnHIF**									
0 nM dFdC/dFdU (N)	49.22	8.46		23.60	4.67		16.97	2.19	
8 nM dFdC (N)	41.89	18.19		31.65	15.59		13.38	7.75	
2 μM dFdU (N)	41.77	18.66	*	44.09	14.32	*	7.94	1.98	
0 nM dFdC/dFdU (H)	47.65	8.00		28.50	5.61	¶	13.03	3.99	¶
8 nM dFdC (H)	40.86	13.01		34.76	14.29		12.90	8.00	
2 μM dFdU (H)	51.22	12.61		34.17	8.23		7.18	1.81	

dFdC: gemcitabine; N: normoxia; H: hypoxia.

¶: p < 0.05 vs. corresponding normoxic condition (same treatment, same cell line); *: p < 0.05 vs. untreated control (same pO_2_, same cell line).

All results are from at least 3 independent experiments ± SD.

## Discussion

The therapeutic implications of oxygen deficiency have been fuelling cancer research for over 100 years. However, detailed studies on the impact of hypoxia on the cytotoxic and/or radiosensitising effects of anticancer drugs are lacking. As the adaptation of tumour cells to hypoxia is primarily mediated by stabilisation of HIF-1, we evaluated the role of functional HIF-1 in the response to chemoradiotherapy. Interestingly, several studies have shown that the presence of HIF-1α is a negative prognostic factor for human breast cancer [21–23]. Animal studies of metastatic breast cancer have demonstrated that lack of HIF-1α in malignant cells significantly reduced tumour progression and metastasis [24]. Moreover, high HIF-1α levels were shown to be predictive of response to epirubicin therapy in patients with breast cancer [25].

In our study, Western blotting showed a consistent upregulation of HIF-1α protein level under hypoxia, whereas the PCR array indicated that HIF-1α was downregulated on mRNA level under hypoxia. Similarly, exposure of HeLa cells to hypoxia (1% O_2_) or the oxygen mimetic CoCl_2_ for 2.5 h did not change HIF-1α mRNA levels significantly, while HIF-1α protein levels increased [26]. In patients with colon cancer, high HIF-1 expression was demonstrated using immunohistochemistry, but no significant difference in HIF-1α mRNA expression between tumour groups and control groups was noticed [27]. One possible explanation for such a discrepancy between mRNA and protein levels is that induction of HIF-1α protein expression is not due to enhanced HIF-1α gene transcription or elevated mRNA stability, but instead results from a longer half-life of the protein due to increased HIF-1α translation and decreased HIF-1α proteolysis [26].

Importantly, due to the above-described transient stabilisation and short half-life of endogenous HIF-1α, HIF targets such as CA9 and the glucose transporter 1 (GLUT-1) have been used to detect hypoxic response in tumour tissues. In breast cancer, abundant expression of CA9 and GLUT-1 was shown to be associated with high-grade cancers and poor prognosis [28, 29]. Moreover, CA9 has also been suggested as a predictive marker for response to doxorubicin treatment and adjuvant endocrine therapy in patients with breast cancer [30, 31]. In addition, several gene and miRNA expression signatures have been described to be associated with poor prognosis in breast carcinoma [32]. In this respect, there is a pressing need for better biomarkers of hypoxia (including gene expression profiles, serum proteins, circulating tumour cells or functional imaging) that could be used non-invasively in patients to enable more rigorous testing of its prognostic/predictive value [33].

Concerning the cytotoxic effect of gemcitabine, no significant influence of hypoxia was observed in the breast carcinoma cell lines included in the present study. In addition, for the first time, the effect of gemcitabine’s main metabolite dFdU was investigated under reduced oxygen conditions and a similar cytotoxic effect was shown under normoxia and hypoxia. This might be explained by the fact that hypoxia has been shown to have no effect on protein expression of several key enzymes (including dCK and cytidine monophosphate kinase) responsible for metabolism of gemcitabine [34].

Moreover, we noticed that both gemcitabine and dFdU induced a clear S phase block in normoxic and hypoxic cells, independent on HIF-1 functionality. Also, no reduction of cellular uptake and DNA incorporation of gemcitabine under hypoxia was reported in pancreatic carcinoma and hepatoma-derived cell lines [35].

Other papers however showed that oxygen deficiency did compromise the cytotoxic effect of gemcitabine, suggesting a cell type dependency of this phenomenon. For example, treatment of transitional cell carcinoma cells with gemcitabine was less effective under hypoxia [36]. For pancreatic cancer cells, several studies reported that hypoxia induced resistance to gemcitabine, by altered signalling through PI3K/Akt/NF-κB pathways and partially through MAPK signalling pathway [37], by reducing both inhibition of proliferation and induction of apoptosis by gemcitabine [38], and by decreasing the synthesis of active gemcitabine deoxynucleotides, possibly also through downregulation of dCK [39]. As such, the impact of tumour-associated hypoxia on the cytotoxic effect of gemcitabine is still not completely resolved.

The present report showed no association between radiosensitisation by gemcitabine or dFdU and HIF-1 functionality. Previous work either focused on the relationship between HIF-1 and the cytotoxic effect of gemcitabine or between HIF-1 and radiosensitivity per se.

Firstly, previous observations have been somewhat controversial regarding HIF-1α expression and the sensitivity to gemcitabine. Suppression of HIF-1α using siRNA resulted in an enhanced efficacy of gemcitabine in the treatment of several pancreatic tumour cell lines [40, 41]. Nevertheless, in line with our results, knockdown of HIF-1α has also been reported to have no effect on the sensitivity of pancreatic PANC-1 cells when treated with gemcitabine under hypoxic conditions [35]. In addition, HIF-1α expression levels after platinum/gemcitabine therapy did not correlate with outcome of patients with stage II/III non-small cell lung cancer and HIF-1α expression was not associated with adverse effects or outcome in patients with pancreatic cancer [42]. As such, the therapeutic value of an approach by which gemcitabine is combined with inactivation of HIF-1α signalling by novel strategies remains to be fully elucidated.

Secondly, the effects of HIF-1 blockade on tumour radiosensitivity are complex. Downstream effects of HIF-1 serve to help tumour cells to adapt to hypoxic stress. In doing so, they change the tumour phenotype in ways that might impact radiosensitivity, some positively and some negatively, but the degree and direction of that influence appears to be dependent on the context. For example, inhibition of HIF-1 activation using siRNA clearly increased radiosensitivity of hypoxic fibrosarcoma cells [43]. Other studies however suggested that HIF-1 would not affect radiosensitivity [44]. From the experimental model used in this study, two conclusions can be drawn. Firstly, no significant difference in radiosensitivity was observed for HIF-1 proficient versus deficient cells, with an OER around 1.50 for all three cell lines. A comparable and relatively low OER of 1.86 ± 0.73 has been reported for MDA-MB-231 by Lagadec *et al.*, who suggested that a negative correlation exists between the OER and increasing malignancy of the breast cancer subtype the cell lines were originally derived from [45]. Secondly, none of the radiobiological parameters (ID_50_, MID, SF_2_) calculated were significantly influenced by HIF-1 functionality after treatment with gemcitabine or dFdU in combination with radiation.

One important limitation of our *in vitro* study is the lack of the microenvironment that would surround tumours *in vivo*. Therefore, further studies using tumour animal models would certainly be warranted. Only in this way, an in-depth understanding and characterisation of hypoxia in breast cancer and other relevant tumour types can be established, ultimately enabling an enhanced prediction of prognosis, optimisation of (gemcitabine and/or radiation) treatment and information on whether and how to target tumour hypoxia.

## Conclusions

Taking into account our previous work in lung cancer cell lines [12], this study showed that the retained radiosensitising effect of gemcitabine under hypoxia was not tumour tissue specific and could be observed in MDA-MB-231 breast cancer cells. For the first time, radiosensitisation by dFdU, the main metabolite of gemcitabine, was investigated and demonstrated under low oxygen conditions. As dFdU has a prolonged half-life, the sustained presence of dFdU in the blood might induce radiosensitisation despite the short half-life of the parent drug gemcitabine. This might be highly relevant, especially considering delivery of the drug to hypoxic tumour regions. As HIF-1 proficient and HIF-1 deficient cells were equally radiosensitised, no major role for functional HIF-1 protein in radiosensitisation by gemcitabine or dFdU could be demonstrated.
